# Clinician Perspectives on the Design and Application of Wearable Cardiac Technologies for Older Adults: Qualitative Study

**DOI:** 10.2196/17299

**Published:** 2020-06-18

**Authors:** Caleb Ferguson, Sally C Inglis, Paul P Breen, Gaetano D Gargiulo, Victoria Byiers, Peter S Macdonald, Louise D Hickman

**Affiliations:** 1 Western Sydney Nursing & Midwifery Research Centre Western Sydney Local Health District and Western Sydney University Blacktown Australia; 2 IMPACCT, Faculty of Health University of Technology Sydney Sydney Australia; 3 MARCS Institute for Brain, Behaviour and Development Western Sydney University Sydney Australia; 4 The Sutherland Hospital South Eastern Sydney Local Health District Sydney Australia; 5 Victor Chang Cardiac Research Institute University of New South Wales Sydney Australia; 6 Heart Lung Clinic St Vincent’s Hospital, St Vincent’s Health Australia Sydney Australia

**Keywords:** technology, arrhythmia, monitoring, older people, cardiology, qualitative, wearable

## Abstract

**Background:**

New wearable devices (for example, AliveCor or Zio patch) offer promise in detecting arrhythmia and monitoring cardiac health status, among other clinically useful parameters in older adults. However, the clinical utility and usability from the perspectives of clinicians is largely unexplored.

**Objective:**

This study aimed to explore clinician perspectives on the use of wearable cardiac monitoring technology for older adults.

**Methods:**

A descriptive qualitative study was conducted using semistructured focus group interviews. Clinicians were recruited through purposive sampling of physicians, nurses, and allied health staff working in 3 tertiary-level hospitals. Verbatim transcripts were analyzed using thematic content analysis to identify themes.

**Results:**

Clinicians representing physicians, nurses, and allied health staff working in 3 tertiary-level hospitals completed 4 focus group interviews between May 2019 and July 2019. There were 50 participants (28 men and 22 women), including cardiologists, geriatricians, nurses, and allied health staff. The focus groups generated the following 3 overarching, interrelated themes: (1) the current state of play, understanding the perceived challenges of patient cardiac monitoring in hospitals, (2) priorities in cardiac monitoring, what parameters new technologies should measure, and (3) cardiac monitoring of the future, “the ideal device.”

**Conclusions:**

There remain pitfalls related to the design of wearable cardiac technology for older adults that present clinical challenges. These pitfalls and challenges likely negatively impact the uptake of wearable cardiac monitoring in routine clinical care. Partnering with clinicians and patients in the co-design of new wearable cardiac monitoring technologies is critical to optimize the use of these devices and their uptake in clinical care.

## Introduction

There is a proliferation in the design, development, and availability of novel consumer-grade wearable and app-based technologies for monitoring cardiac rate and rhythm. Yet, the optimal method and duration of monitoring remains unknown [1]. New devices include technologies such as AliveCor (a smartphone-based device with functionality to record a single point in time; a single-lead electrocardiogram) and Zio patch (a wearable adhesive patch to monitor heart rhythm for a prolonged duration). There is a growing demand for new health-monitoring technologies to assist clinicians in diagnosis, clinical decision making, treatment, and ongoing management of older adults [2]. This demand is driven by caring clinicians and by public interest in new technologies. Advanced age is a key risk factor in the development of heart failure, atrial fibrillation, and stroke, with a dramatically increased risk over the age of 80 years [3-5]. Novel wearable monitoring devices offer promise in detecting arrhythmia and monitoring cardiac health status, among other potentially clinically valuable parameters. Wearables are revolutionizing health care delivery, yet it is difficult ascertain the number of health problems that these technologies may help to intervene and contribute to the provision of quality care [6]. Capabilities may include physiological and biochemical sensing, as well as motion sensing, which can be applied for diagnostic and ongoing monitoring [6,7]. Physiological monitoring by wearables could help in the diagnosis and treatment of a large number of individuals with cardiovascular, neurological, and pulmonary diseases. Further, home-based motion sensing might help prevent falls and maximize an individual's independence and community participation [8]. Wearables aim to improve quality of care and support health systems by triggering an alert based on abnormal parameters. They can aid in the diagnosis and treatment of illnesses in a timely and efficient manner. In particular, wearable technologies are increasing in popularity among cardiac patients, rehabilitation patients, and older patients. Due to the rapid pace of innovation, it is important to ensure that these technologies are suited to the individual needs of older adults. However, the clinical utility and usability from the perspectives of clinicians is largely unexplored. It is critical to explore the factors that impact translation from bench to bedside, upscale and sustainability of new devices in clinical practice. Further, there is a need to explore the use of cardiac monitoring devices to improve the detection and management of cardiovascular conditions and contribute to the improvement of the quality of life in older adults.

### Aim

This study aimed to explore clinician perspectives on the use of wearable cardiac monitoring technology for older adults.

The 3 key objectives were (1) to explore clinical issues with current monitoring challenges, including barriers to use and uptake by older adults; (2) to explore priorities for the development of future technologies and identify the parameters of clinical importance; and (3) to explore the design of an “ideal device” for cardiac monitoring in older people.

## Methods

### Study Design

A descriptive exploratory qualitative design with semistructured focus groups involving clinicians was used.

### Participant Selection

A convenience sampling technique was used to select study participants. Physicians, nurses, and allied health professionals working in tertiary-level hospitals who were available on the day of data collection were invited to participate in the face-to-face focus group discussion. Staff participated in a group that suited the daily routine of the clinical setting.

### Setting

Physicians, nurses, and allied health professionals working in cardiology, rehabilitation, cardiac rehabilitation, and aged care services, at 3 major tertiary-level hospitals in Sydney, Australia, were invited to participate between May 2019 and July 2019.

### Data Collection

Two expert facilitators, CF and LDH, conducted the focus groups. Both researchers are skilled in qualitative research methods and focus group facilitation. Prior to commencement of the focus group session, the facilitator highlighted the purpose of the group and outlined roles and responsibilities. Data were collected using audio recording, combined with field notes made at the time of the focus group interviews. Focus group sessions lasted 30-60 minutes to facilitate the conversation and reach data saturation. An interview guide was used to guide discussions. Probing questions were used when required by the facilitator to ascertain further information.

### Interview Guide

The following questions were used to guide the focus group discussions:

1. Can you tell me about your experiences of cardiac monitoring in older people?
2. What are the issues with current cardiovascular monitoring technologies?
3. Why would this technology need to be replaced?
4. What clinical data or parameters do you want to measure in older people?
5. What health-related data do you want to capture when caring for older people?
6. How do you want the data to be presented or fed back to you?
7. What else would you like to know?
8. What is one bit of information that you would like to know that you can’t get now?

### Data Analysis

All focus group interviews were transcribed verbatim by research interns, and the transcripts were coded by 3 members of the research team. The data coders systematically read, searched, coded, and arranged. The Braun and Clarke [9] method of thematic analysis was used, and codes were clustered into groups before identification of any themes. Reducing the huge amounts of raw data from codes into categories and themes was an iterative process, whereby the codes were subsequently assigned into categories before finalizing the overarching themes. Authors and members of the interviewee team were involved in the data analysis to ensure rigor and accuracy of the analysis and consensus.

### Ethical Considerations

This study was approved by the Western Sydney Local Health District (Human Research Ethics Review Ref LNR/18/WMEAD/513) and received Western Sydney University external recognition approval (Human Research Ethics Review Ref H13228). This study was conducted in compliance with the principles of the Declaration of Helsinki [10]. Focus groups were conducted in a confidential area. Written informed consent was obtained from all participants. The funders of this research were not involved in the study design, data collection, analysis, or interpretation, and were not involved in the publication of the final manuscript.

## Results

### Principal Findings

A total of 4 focus groups were completed (Table 1). Participants represented physicians, nurses, and allied health staff working in 3 tertiary-level hospitals. All 4 focus group interviews were completed between May 2019 and July 2019. There were 50 participants, 22 female and 28 male. A diversity of professions was represented, including 5 cardiologists, 4 geriatricians, 10 allied health professionals, 15 junior and resident medical officers and students, and 16 nurses.

**Table 1:** Baseline demographics of participants.

Focus group	Setting	Participants	Occupation
Group 1	Rehabilitation	n=13 (11 female, 2 male)	Nurses
Group 2	Cardiology and heart failure service	n=25 (4 female, 21 male)	Cardiologists Medical students Medical officers Allied health
Group 3	Cardiac rehabilitation service	n=6 (4 female, 2 male)	Allied health Nurses
Group 4	Aged care and rehabilitation	n=6 (3 female, 3 male)	Allied health Geriatricians Nurse
Total		n=50 (22 female, 28 male)	

The following 3 themes emerged that illustrated clinician perspectives on the use of wearable cardiac monitoring technology for older adults.

1. The current state of technology—understanding the perceived challenges of patient cardiac monitoring in hospitals
2. Priorities in cardiac monitoring—what new parameters could be clinically helpful
3. The ideal device—cardiac monitoring of the future

#### Theme 1: The Current State of Technology—Understanding the Perceived Challenges of Patient Cardiac Monitoring in Hospitals

This theme reflects the current challenges faced by clinicians in hospitals and the perceived areas of improvement in designing new technology for cardiac monitoring in older people. Three subthemes were clearly identified.

##### Subtheme 1: Physical Form and Function of Device

This subtheme includes all aspects of device structure and use that lead to negative experiences by either the user or the clinician. These aspects were as follows:

- Reduced accuracy of recorded data due to lead disconnection, movement interference, lack of continuous readings, interference, and false data
- Delirious patients pulling leads off
- Difficulty in use due to user not being technologically advanced
- Beeping causing anxiety and provoking panic

It’s annoying because it’s connected and it’s got a wire. They don’t read particularly well if they get loose.

Focus group 3, cardiac rehabilitation professionals

A lot of data that the systems currently record is not real or not useful, so lots of interference from leads.

Focus group 4, aged care professionals

A lot of our patients are older—anything too techy can get too frustrating.

Focus group 1, rehabilitation nurses

People freak out when they hear beeping.

Focus group 4, aged care professionals

##### Subtheme 2: Wearability of the Device

In terms of overall device wearability, patients do not like heavy devices around the neck, nor do they like leads and adhesives, as these get caught and feel restrictive. Concern was expressed about potential device-related skin and pressure injuries, hygiene, and infection control issues. In terms of device type selection, single-use and disposable devices were seen as wasteful and not ecologically friendly. It was also reported that hospitals often purchase cheap devices with short usable lifespans. “We don’t need the dots and the leads and it’s quite heavy. Confused patients want to rip everything off.” (Focus group 4, aged care professionals.)

##### Subtheme 3: Device Data Management

Groups reported difficulty in interpreting the data produced, data overload, and alert fatigue. Questions were raised over ownership of data. “We just pick the parameters we would like and not have to use all the data available.” (Focus group 4, aged care professionals.)

#### Theme 2: Priorities in Cardiac Monitoring—What New Parameters Could Be Clinically Helpful

There was consensus from all clinicians for preference to select continuous monitoring over intermittent, where possible. These included blood pressure, pulse rate, heart rhythm, glucose level, oxygen saturation, mobility, and fluid status. The considerations around the best ways in which to provide these data to clinicians varied depending on the clinician role.

Continuous accurate data. It might actually help us find out why they are falling.

Focus group 1, rehabilitation nurses

Patients are very individual and that ability to tailor that to that individual and their circumstances which are unique.

Focus group 4, aged care professionals

If they are on fluid restriction…they could be having sneaky drinks on the side, you can’t keep track of exactly what their input is.

Focus group 1, rehabilitation nurses

#### Theme 3: The Ideal Device—Cardiac Monitoring of the Future

The clinicians responded well to the idea of having input into what the ideal device would look like. The findings generated the following 2 core areas of consideration: (1) form, wearability, and characteristics, and (2) functionality.

##### Consideration 1: Form, Wearability, and Characteristics

The physical form of suggested technology included more obvious solutions, such as a watch, The physical form of suggested technology included more obvious solutions, such as a watch, tracker, iPad, smartphone, computer with internet page with a code linking to a device, sweat patches, mattress, and cushion-based technology. It appeared difficult for clinicians to think outside of the current health system parameter for design, which limited thinking at times. Sometimes it was challenging to generate thinking beyond the design of currently available devices. Wearability and characteristics were often described in single-word terms, such as comfortable, Velcro, waterproof, cleanable, small, lightweight, noninvasive, no beeping, wide and elasticized, alarm feature, wireless, easy to access and apply, and user-friendly.

Ideally nothing attached…no wires, no electrodes. Can shower in it. Easy to attach.

Focus group 4, aged care professionals

Something quick, on the wrist.

Focus group 3, cardiac rehabilitation professionals

Not too technologically advanced. Not wasteful. Hygienic.

Focus group 1, rehabilitation nurses

##### Consideration 2: Functionality

Clinicians described functionality using phrases such as ability to interpret data and information to provide action, specific and tailored to individual patient needs, continuous real-time data to monitor change over time, flexibility in adjusting and setting own parameters and modify medications, communication fed back to patients, physical activity tracking, and ability to access data anywhere and anytime.

Change the parameters, so you aren’t going to constantly get alerts you don’t need. Something that would alarm to let you know. Reminder if you’ve not moved in a while, reminder to have a drink—passive prompts.

Focus group 1, rehabilitation nurses

Kind of alarm setup, so if their stats dropped below a certain range or that their blood pressure was going up, down, heart rate’s going up. Show a visual picture…show them this is what’s happening, this is where you are, this is where you need to be.

Focus group 3, cardiac rehabilitation professionals

## Discussion

Findings generated the following 3 overarching, interrelated themes: (1) the current state of play, understanding the perceived challenges of patient cardiac monitoring in hospitals, (2) priorities in cardiac monitoring, and what parameters new technologies should measure, and (3) cardiac monitoring of the future, the “ideal device.”

Theme 1 elucidated the flawed design with current cardiac monitoring technology and how this inhibits gaining the full potential value of the best available data to inform patient care. Fundamental to this theme was the physical form and function of the device, and secondly, the management of data captured by the device [11].

A large proportion of cardiac monitoring devices used in hospitals and outpatient settings, such as Holter monitors, do not reflect the latest available technology; these technologies are frequently outdated relative to our personal technology at home [12,13]. The gap between commercially available technology and health service provision of technology is wide and could widen as the speed at which new consumer technologies entering the market increases [14].

Clinicians expressed frustration over the bulky and unattractive nature of the devices currently used in clinical settings, especially when comparing these devices with their own smartwatch or smart devices at home [15]. Adhesive skin dots, stiff cords, and a heavy, awkward battery pack may have never been that acceptable to patients, but they were tolerated relatively well at a time when this was the only method in which to collect and monitor such data [16].

Just as the physical and tactile design of health and monitoring technology has improved significantly, the way in which we receive, view, and are informed of data has changed immeasurably [14]. Our personal devices provide us with attractive curated data, which, due to artificial intelligence and machine learning, has already filtered out unnecessary data and errors, such as artifacts. We are presented with graphical summaries and pictorials of what we need to know [17]. Therefore, when busy clinicians log on to view data from a device in clinical practice, the sense of frustration and dissatisfaction increases when they are faced with errors, artifacts, and missing or incomplete data [12].

Theme 1 is supported by literature that clinicians favor devices with a physical form that is unobtrusive and attractive, yet practical in terms of hygiene and affordability; they also favor a platform and system that are easy to use and that they have confidence in with respect to data management (privacy as well as the alerts generated from the data) [18,19].

Clinicians value how data collected from patients can inform their clinical practice [20]. Interestingly, clinicians value collecting data from several parameters, with no push to limit data collection from only one or two parameters. It appears that more parameters are better, providing relevant, accurate, and comprehensive understanding of a patient’s condition [21]. This feature may add to design challenges for devices as they are expected to measure several physiological parameters, which could result in a physical design that is less streamlined than might be achievable with fewer parameters [22].

The future of health care and technology design requires multiple touch points from bench research to bedside patient use if future design is going to truly revolutionize care; it must meet the needs of users and support health professionals in their work [23]. Health data that are derived from wearable devices can frequently suffer from irregularities that affect the overall usefulness in care decision making and delivery [11]. Challenges of the current wearable technologies include lack of capacity to generate specific and accurate data, which could lead to anxiety and panic from the perspectives of the patient and the provider [19]. Wearable technologies can suffer from reliability problems [24], so ensuring accuracy is important for the generation of future data [25]. Interventions that are based on inaccurate data could put patient safety in jeopardy by inducing medication or procedural errors. Current technologies have some features that undermine patient comfort [14]. The benefit of these technologies could be limited if patients do not find them comfortable. Future technologies should prioritize the comfort of patients [15]. Wearable technologies could affect the physical, psychological, and social aspects of a patient’s life [26]. Heavy devices, beeping, and devices that are difficult to clean could negatively affect user experience and sustainability of use [27]. Health care technologies are expensive. As a result, hospitals tend to buy cheaper devices that lack quality and have shorter lifespans [28]. Another critical challenge related to wearable technology is a lack of capacity to generate appropriate information that is unique to the patient [29].

Protecting patient health information, including medical and physiological data, is a major ethical obligation of health professionals and health care systems. Easy access to data generated from wearable technologies could lead to misuse of sensitive medical data [18,19]. These technologies also lack the capacity to interpret and make sense of the data for further action [30]. This could keep patients dependent on health professionals for situations that patients could have resolved themselves.

Theme 2 highlights the essential design priorities in cardiac monitoring and the parameters that new technologies should measure. Form, functionality, wearability, and characteristics were highlighted as essential features for designers to consider (Multimedia Appendix 1). It has been recommended that emerging health technologies include features that can be tailored or individualized to a patient’s condition [15,31]. Different patients have different cardiovascular conditions, and these wearable technologies should be capable of tailoring to each patient’s uniqueness [21]. Health care professionals would also favor wearable technologies that can generate continuous and accurate data [20]. The ability to capture multiple parameters was preferred over single-parameter monitoring devices.

Theme 3 explored “the ideal device.” The development of next-generation devices should include an iterative design with clinicians, patients, and end users. It was recognized by the groups that current cardiac monitoring technologies are heavy, uncomfortable, connected to wires, and easily damaged [12,13]. These technologies affect physical, psychological and social aspects of a patient’s life, elements which should be considered in the development process [24,32-34]. The groups identified key recommendations for future devices. Health care professionals would prefer the ideal cardiac monitoring to be more comfortable, wireless, waterproof, and user friendly [35,36]. Wearable technologies with a capacity to provide accurate data continuously are highly valued by health care providers [15,37]. Our findings were similar to other research that highlights factors, including user-friendliness, satisfaction with design, comfort, and motivation, to be the important factors to enhance uptake [38]. It is important to tailor any monitoring solutions to meet the needs of individual patients, recognizing that one size does not fit all [38]. This is of particular importance for longer-term users, to enhance adherence to monitoring and wearability.

### Conclusion

Existing wearable cardiac monitoring technologies for older adults do not fully address the needs of clinicians and their patients. A range of improvements are desirable to ensure these technologies have minimal impact on the patient (physically, psychologically, and socially). Substantial improvements in information provided by the device are desired. These improvements include the number of physiological parameters collected, reliability of data quality, continuity of data, capability of customizing data to individual patients, and a means of presenting data in an intelligible form that can impact patient care efficiently. These and other challenges will directly impact uptake in routine clinical care. Future acceptance of new wearable devices will rely on functionality and design for comfort as well as clinical accuracy. These must be considered early in the development process. Partnering with clinicians and patients in the co-design of new wearable cardiac monitoring technologies is critical to optimize the use of these devices and their contribution to patient care.

## Appendix

Multimedia Appendix 1 Themes.
