# Supplementary material for: Clinician Perspectives on the Design and Application of Wearable Cardiac Technologies for Older Adults: Qualitative Study
Source: JMIR Aging. 2020 Jun 18;3(1):e17299. doi: 10.2196/17299 (PMC7333070; doi:10.2196/17299)
Supplement: Multimedia Appendix 1 [file aging_v3i1e17299_app1.docx]

| **Themes** |  | Quotes |
| --- | --- | --- |
| **Current Challenges** | - Patients forgetting to put the device on - Disconnection from leads: incorrect readings, interference and false data - Heavy device around neck - Confused patients pulling leads off - Patients do not like leads and adhesives as they get caught and feel restricted - Hospitals with cheap devices and short life - Data overload - Beeping is anxiety and panic provoking | - “We don’t need the dots and the leads and its quite heavy” - “It’s annoying cos its connected and it’s got a wire … they don’t read particularly well if they get loose” - “a lot of data that the systems currently record is not real or not useful, so lots of interference from leads” - “People freak out when they hear beeping” |
| **Ideal Device** | Form: watch, iPad, phone, computer with internet page with a code linking to a device, sweat patches, mattress and cushion based technology  Wearability: comfortable, ease of access and applicability  Characteristic: Velcro, user friendly, waterproof, cleanable, small, lightweight, non-invasive, no beeping, wide and elasticised, alarm feature and wireless  Functionality:   - Ability to interpret data and information to provide action - Specific and tailored to each patient needs - Continuous real-time data to monitor change over time - Flexibility in adjusting and setting own parameters and modify medications - Communication fed back to the patients - Physical activity tracking - Access data anywhere and anytime | - “…having to put leads on might be too difficult … something quick, on the wrist” - “Ideally nothing attached. No wires, no electrodes. Can shower in it. Easy to attach” - Continuous data - “it might actually help us find out why they are falling” - “…change the parameters, so you aren’t going to constantly get alerts you don’t need” - “something that would alarm to let you know …reminder if you’ve not moved in a while, reminder to have a drink – passive prompts” - “…kind of alarm set up so if their stats dropped below a certain range or that their blood pressure was going up, down, heart rate’s going up...” - “show a visual picture … show them this is what’s happening, this is where you are, this is where you need to be” - “Patients are very individual and that ability to tailor that to that individual and their circumstances which are unique” |
| **Potential problems for ideal device** | - Ownership of data - Data overload and alert fatigue - Hygiene and infection control issues - Not too technologically advanced - Pressure injuries - Single use and disposable is wasteful - Interpreting the data | - “A lot of our patients are older - anything too techy can get too frustrating” - “confused patients want to rip everything off” - “We just pick the parameters we would like and not have to use all the data available” |
| **Parameters to measure** | - Blood pressure - Pulse rate - Heart rhythm - Glucose level - SpO2 - General vitals - Mobility - Fluids | - “If they are on fluid restriction …they could be having sneaky drinks on the side, you can’t keep track of exactly what their input is” |
